# Unraveling the potential co-contributions of cerebral small vessel vasculopathy to the pathogenesis of Alzheimer’s dementia

**DOI:** 10.1186/s13195-015-0133-2

**Published:** 2015-07-10

**Authors:** Jodi D. Edwards, Joel Ramirez, Sandra E. Black

**Affiliations:** LC Campbell Cognitive Neurology Research Unit, Hurvitz Brain Sciences Research Program, Sunnybrook Research Institute, Toronto, ON M4N 3M5 Canada; Heart & Stroke Foundation Canadian Partnership for Stroke Recovery, Sunnybrook Health Sciences Centre, Toronto, ON M4N 3M5 Canada; Institute of Medical Science, University of Toronto, Toronto, ON M5S 1A8 Canada; Department of Medicine, Neurology, Sunnybrook Health Sciences Centre, Toronto, ON M4N 3M5 Canada

## Abstract

Emerging evidence for the potential co-contributions of small vessel vasculopathy to dementia has resulted in a more nuanced view of Alzheimer’s disease (AD) pathogenesis. Although cerebral small vessel disease, visualized on magnetic resonance imaging as hyperintense signal abnormalities, independently predicts the incidence and clinical progression of dementia, the relationships between AD pathology, white matter hyperintensity volume, genotype, and cognitive decline in AD remain unclear. The study by Morgen and colleagues, recently published in *Alzheimer’s Research & Therapy,* presents important new findings on the associations between apolipoprotien E ε4 genotype, white matter hyperintensities, and cognition, independent of vascular risk, in a cohort of AD patients.

Alzheimer’s disease (AD) is considered the most common pathology contributing to dementia. Although genomic analyses have identified the apolipoprotein E (apoE) ε4 allele as a major risk factor for late-onset AD, cerebral small vessel disease (SVD) may also play a significant role in decline to dementia. Despite increased efforts to elucidate the complex relationships between AD, SVD, and apoE genotype, the evidence remains equivocal, posing a challenge for understanding the pathogenic mechanisms underlying aging and dementia.

Prevailing models of AD pathogenesis suggest that the aggregation of oligomeric amyloid-beta (Aβ) proteins initiates a pathophysiological cascade hallmarked by extracellular Aβ plaques in the interstitium and along cerebral vessels, and intraneuronal neurofibrillary tangles of hyperphosphorylated tau. Neurodegenerative sequelae include synaptic dysfunction, cortical atrophy, and progressive cognitive decline. In vivo models of AD pathogenesis have identified several potential mechanisms by which apoE ε4 may contribute to amyloidogenic processes (Fig. 1) [1].

**Fig. 1:**
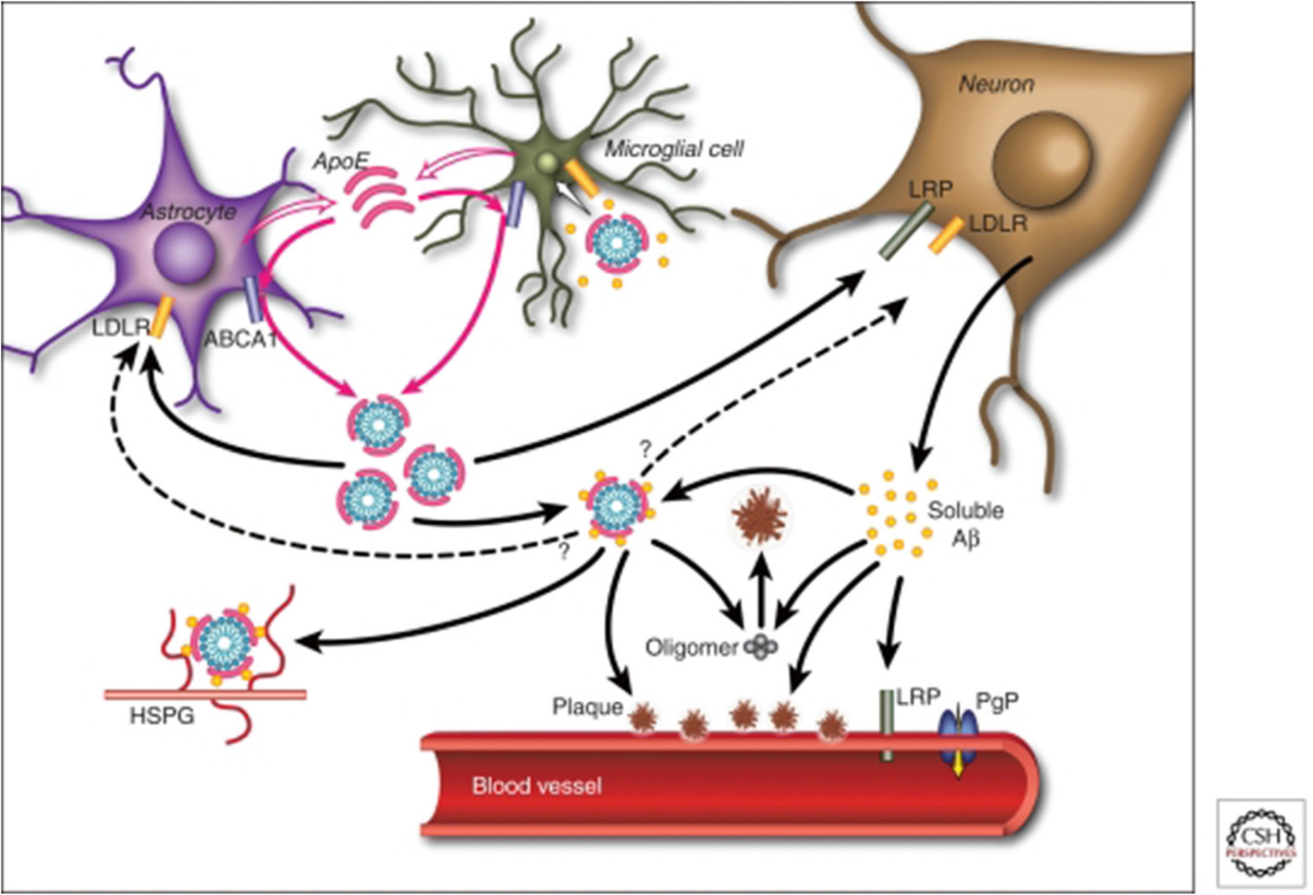
Effects of apolipoprotein E (apoE) ε4 on amyloid-beta (Aβ) metabolism and deposition. apoE may facilitate cellular uptake of Aβ by the endocytosis of apoE-containing lipoprotein particles bound to soluble Aβ (yellow circles), or via binding of this apoE-Aβ complex (mauve-blue circles) to extracellular heparin sulfate proteoglycans (HSPG). Alternatively, apoE may impair Aβ clearance by slowing the transport of Aβ across the blood–brain barrier [1]. LDLR, low-density lipoprotein receptor; LRP, lipoprotein receptor-related protein; PgP, permeability-glycoprotein. Reproduced with permission from Elsevier [1]

In recent decades, a more nuanced view of AD pathogenesis has emerged, with evidence for the putative co-contributions of vascular pathology. SVD is commonly observed at autopsy in patients with AD and, although neuropathological studies have consistently shown additive, not interactive effects of vasculopathy [2], white matter hyperintensities (WMHs) are more difficult to capture at end-stage disease. Several neuroimaging-derived biomarkers for SVD have been identified, including periventricular and deep WMHs of presumed vascular origin, lacunes, and enlarged perivascular Virchow-Robin spaces, which may be visualized earlier in disease progression [3]. Recent studies using amyloid positron emission tomography (PET) support a relationship between SVD and amyloid pathology [4], but also confirm that significant subcortical ischemic vasculopathy alone may be sufficient to cause dementia [5]. Combined with early clinical evidence for the role of apoE ε4 in the development of cerebral amyloid angiopathy independent of AD [6], and observational associations between dementia and vascular risk factors, these advances have initiated new lines of investigation into whether white matter vasculopathy represents a synergistic or independent mechanism of AD pathogenesis.

In a recent article in *Alzheimer’s Research & Therapy*, Morgen and colleagues [7] retrospectively examined relationships between apoE genotype, WMHs, and cognitive performance in a cohort of patients with mild to moderate AD (N = 183). They compared apoE ε4 carriers to non-carriers with respect to global and regional WMH volume and performance on standardized neuropsychological tests. Results indicated that apoE ε4 carriers showed reduced WMH volumes compared with non-carriers and, among non-carriers, global WMH volume was correlated with performance on the Trail Making Test-A of executive function. The authors interpreted these findings as evidence that WMHs had a functional impact on cognition and that SVD represents an independent mechanism of AD pathogenesis.

The Morgen et al. study is consistent with prior work demonstrating that WMHs primarily impact the domain of executive function [8] and are associated with amyloid plaques on Pittsburg compound B retention PET in apoE ε4 non-carriers with subcortical vascular cognitive impairment [9]. In a recent meta-analysis of 42 studies, however, an association between apoE ε4 and magnetic resonance imaging markers of cerebrovascular disease was reported [10], suggesting that vascular mechanisms may differentially co-contribute to multiple pathophysiological cascades with distinct clinical sequelae.

A strength of the Morgen et al. study [7] is in the specificity of case selection. To minimize the potential for misclassification, the authors excluded those with ‘severe’ WMH burden suggestive of vascular dementia according to new AD diagnostic guidelines [11]. They then adjusted all multivariate models for multiple vascular risk factors, to increase the likelihood of observing apoE-dependent effects. In restricting their cohort to patients without a presumed vascular etiology, Morgen et al. provide novel evidence for an association between WMH and apoE genotype that modified executive function independent of vascular risk.

A major source of bias in clinical imaging studies is that template-matching approaches, such as statistical parametric mapping, do not include a head size correction or account for cortical atrophy. In many clinical populations, particularly AD, the degree of atrophy is high, impacting ventricle size and total intracranial volume and resulting in the potential misattribution of WMH to the grey matter tissue compartment [12]. To partially address this limitation, Morgen et al. corrected for total intracranial volume and still showed greater WMH burden in apoE ε4 non-carriers, strengthening their argument that this association reflects pathogenic mechanisms of AD independent of apoE ε4-mediated neurodegeneration.

As arterial hypertension correlated with WMHs in their cohort, Morgen et al. suggest blood pressure may have synergistic effects on amyloid-mediated endothelial damage. Recent animal studies have described a 'G-lymphatic' system that removes amyloid in the perivascular spaces toward the deep periventricular veins [13] and confirmed an association between arterial hypertension and venous collagenosis in spontaneously hypertensive rats [14]. Neuropathological analyses also suggest that periventricular WMHs may represent perivenular vasogenic edema correlating with collagenosis of the deep medullary venules [15]. Venous insufficiency thus represents a potential mechanism of impaired Aβ clearance that may exacerbate amyloid accumulation and increase clinical decline into dementia.

Increasing evidence for the role of small vessel vasculopathy in dementia highlights the importance of multifactorial mechanisms in AD pathogenesis. The findings of Morgen and colleagues are timely and offer support for the co-contributions of WMHs to cognitive impairment in AD, not mediated by apoE ε4 genotype. A greater understanding of amyloid clearance will be required for the development of targeted therapies to mitigate the risk and clinical progression of AD. Amyloid PET imaging offers new opportunities to elucidate additive/interactive effects of WMHs in AD and unravel the pathogenic mechanisms of this complex phenotype.

## Abbreviations

AD: Alzheimer’s disease
apoE: apolipoprotein E
Aβ: Amyloid-beta
PET: Positron emission tomography
SVD: Cerebral small vessel disease
WMH: White matter hyperintensity
